# Retraction: Chamaejasmin B decreases malignant characteristics of mouse melanoma B16F0 and B16F10 cells

**DOI:** 10.3389/fonc.2026.1858355

**Published:** 2026-04-30

**Authors:** Frontiers Editorial Office

Following publication, concerns were raised about the integrity of the figures included and the ethical oversight of the animal experiments in this study. The authors acknowledged that tumor sizes exceeded internationally accepted animal welfare limits and the parameters described in the ethics approval. In line with Frontiers’ policies, an investigation was conducted and confirmed that, contrary to the statements in the article and the approval documentation, the experiments do not adhere to what had been approved by the Shihezi University Animal Care and Use Committee. This represents a breach of Frontiers’ guidelines and those of the Committee on Publication Ethics. The article is therefore being retracted.

This retraction was approved by the Chief Editors of Frontiers in Oncology and the Chief Executive Editor of Frontiers. The authors agree to this retraction.

Frontiers would like to thank Elisabeth M. Bik for contacting the journal regarding the published article.

